# A *bla*_OXA-181_-harbouring multi-resistant ST147 *Klebsiella pneumoniae* isolate from Pakistan that represent an intermediate stage towards pan-drug resistance

**DOI:** 10.1371/journal.pone.0189438

**Published:** 2017-12-08

**Authors:** Fouzia Nahid, Rabaab Zahra, Linus Sandegren

**Affiliations:** 1 Department of Microbiology, Quaid-I-Azam University, Islamabad, Pakistan; 2 Dept. of Medical Biochemistry & Microbiology, IMBIM, Uppsala University, Uppsala, Sweden; Cornell University, UNITED STATES

## Abstract

Carbapenem resistant *Klebsiella pneumoniae* (CR-KP) infections are an ever-increasing global issue, especially in the Indian subcontinent. Here we report genetic insight into a *bla*_OXA-181_ harbouring *Klebsiella pneumoniae*, belonging to the pandemic lineage ST147, that represents an intermediate stage towards pan-drug resistance. The CR-KP isolate DA48896 was isolated from a patient from Pakistan and was susceptible only to tigecycline and colistin. It harboured *bla*_OXA-181_ and was assigned to sequence type ST147. Analysis from whole genome sequencing revealed a very high sequence similarity to the previously sequenced pan-resistant *K*. *pneumoniae* isolate MS6671 from the United Arab Emirates. The two isolates are very closely related with only 46 chromosomal nucleotide differences, 14 indels and differences in plasmid content. Both carry a substantial number of plasmid-borne and chromosomally encoded resistance determinants. Interestingly, the two differences in susceptibility between the isolates could be attributed to DA48896 lacking an insertion of *bla*_OXA-181_ into the *mgrB* gene that results in colistin resistance in MS6671 and SNPs affecting AcrAB efflux pump expression likely to result in tigecycline resistance. These differences between the otherwise very similar isolates indicate that strong selection has occurred for resistance towards these last-resort drugs and illustrates the trajectory of resistance evolution of OXA-181-producing versions of the ST147 international risk clone.

## Introduction

Over the last two decades carbapenems have emerged as last line of effective therapy for treating the worldwide disseminated extended-spectrum β-lactamase (ESBL)-producing *Enterobacteriaceae*. Various studies from Pakistan have reported prevalence of ESBL-producing *Klebsiella pneumoniae* ranging from 26% to 59% during 2002 to 2013 [1–4]. Hence there is a significantly increasing dependence upon carbapenems for treating such MDR *K*. *pneumoniae*. Although carbapenemases in *Enterobacteriaceae* were relatively rare a decade ago, their emergence and rapid dissemination is now raising concern in health communities all over the globe [5]. Until 1990’s, carbapenemases were considered species-specific and a problem of clonal spread, but in the past decade the interspecies dissemination of carbapenemases has made it a global issue [6]. Hence it is of great significance to understand the genetic context of carbapenemases and how carbapenemase-producing strains further evolve resistance to the last remaining active antibiotics such as colistin. Carbapenemases including VIM, IMP, OXA-48, KPC and NDM-1 have been reported from Pakistan recently [7–9]. However, the knowledge about the variants of these acquired carbapenemases and their genetic contexts still remain scarce.

OXA-181, differing by 4 amino acid changes from OXA-48 while sharing the same hydrolytic properties against carbapenems, has been identified in enterobacterial isolates from India and from patients with a link with the Indian subcontinent [10, 11]. Since the identification of OXA-181 in India in 2007, OXA-181-producing *Enterobacteriaceae* have been reported from several other countries i.e. Bangladesh, Sri Lanka, Nepal, Oman, South Africa, Canada, USA, Australia, France and the United Kingdom [12–21]. Certain *K*. *pneumoniae* sequence types are particularly important in the spread of carbapenemases. Recently, *K*. *pneumoniae* ST147 has been associated with the carriage of OXA-181 in isolates from UAE, Abu Dhabi and USA [22–24]. Here we report and genetically characterize the first carbapenem-resistant ST147 *K*. *pneumoniae* clinical isolate harbouring *bla*_OXA-181_ from Pakistan.

## Materials and methods

### Bacterial isolation and identification

Strain DA48896 was received at the microbiology laboratory of the tertiary hospital Pakistan Institute of Medical Sciences, Islamabad as part of a larger study of carbapenemase producing *K*. *pneumoniae* at the hospital. It was identified as *Klebsiella pneumoniae* using Gram stain and standard biochemical tests including Oxidase test, SIM (Sulfur, Indole, Motility) test, TSI (Tripple sugar iron) test and Simmon citrate agar test.

### Antibiotic susceptibility profiling

The Etest method (Biomerieux, France) was used to determine MIC against routinely used antibiotics according to the manufacturer’s instructions. For colistin the broth dilution method was used according to the protocol of CLSI. The susceptibility results were interpreted according to EUCAST (the European Committee on Antimicrobial Susceptibility Testing) guidelines (www.eucast.org).

### Detection of carbapenemase encoding genes

252 carbapenem resistant *K*. *pneumoniae* isolated during April- September 2015 were screened for the presence of acquired carbapenemase encoding genes including *bla*_IMP_, *bla*_VIM_, *bla*_NDM_, *bla*_SPM_, *bla*_AIM_, *bla*_DIM_, *bla*_GIM_, *bla*_SIM_, *bla*_KPC_, *bla*_BIC_, *bla*_OXA-48_ according to a protocol described previously [25]. Reaction I included primers for *bla*_IMP_, *bla*_VIM_ and *bla*_SPM_. Reaction II included primers for *bla*_NDM_, *bla*_KPC_ and *bla*_BIC_. Reaction III included primers for *bla*_AIM_, *bla*_DIM_, *bla*_GIM_ and *bla*_SIM_. The PCR program consisted of following conditions in T3000 Thermocycler 48 (Biomerta, Germany), initial denaturation at 94°C for 10min, followed by 36 cycles of DNA denaturation at 94°C for 30sec, primer annealing at 52°C for 40sec and primer extension at 72°C for 50sec. The final extension was performed at 72°C for 5min.

### Whole-genome sequencing and bioinformatics

Genomic DNA for whole genome sequencing was prepared using MasterPure^™^ DNA Purification Kit (Epicentre Technologies, Madison, Wisconsin). Pacific Biosciences sequencing was performed at the Science for Life laboratories sequencing platform at Uppsala University using Pacific Biosciences II technology and Illumina MiSeq was performed in-house using the NexteraXT technology. CLC Genomic Workbench v 10 (CLC Bio/Qiagen) with Microbial Genomics Module and Microbial Genome Finishing Tools was used for *de novo* assembly of reads and of reference assembly and variance analysis to *K*. *pneumoniae* LN824133. The sequence data including raw sequence reads and assembled contigs of the chromosome and plasmids have been deposited at NCBI with accession numbers CP024429-CP024436 under BioProject PRJNA348457.

The contigs were submitted to the ResFinder and PlasmidFinder databases at the Centre of Genomic Epidemiology (www.genomicepidemiology.org) to identify antibiotic resistance genes and plasmid replicons [26–28]. MLST was performed by submitting contigs at Institut Pasteur MLST Databases (http://www.pasteur.fr/mlst/).

## Results

In June 2015, a carbapenem resistant *K*. *pneumoniae* was isolated from the tracheal secretion and an endotracheal tube of a 32-year-old female patient at the intensive care unit at the tertiary hospital, Pakistan Institute of Medical sciences, Islamabad. Isolate DA48896 was found to be multi-drug resistant and sensitive only to Tigecycline and Colistin (Table 1). The isolate carried a *bla*_OXA-48_-like carbapenemase encoding gene 100% identical to *bla*_OXA-181_. This was the only OXA-181 producing isolate identified out of 252 screened *K*. *pneumoniae* at the hospital during the study period.

**Table 1:** MICs against routinely used antibiotics.

Antimicrobial Class	Antimicrobial agent	DA48896 MIC (mg/L)	EUCAST interpretation	MS6671 MIC (mg/L)
Carbapenems	Meropenem	8	R	8
	Ertapenem	>32	R	>32
	Doripenem	6	R	4
	Imipenem	24	R	4
Penicillin	Ampicillin	>256	R	>256
Cephalosporins	Cefuroxime	>256	R	>256
	Cefaclor	>256	R	-
	Cefpriome	>256	R	-
	Cefepime	>256	R	32
	Ceftazidime	>256	R	-
Penicillin and beta-lactamase inhibitor	Amoxycillin/clavulanate	>256	R	>256
	Piperacillin/Tazobactam	>32	R	>256
Fuoroquinolones	Ciprofloxacin	4	R	>32
	Levofloxacin	4	R	-
Tetracyclines	Tetracycline	>256	R	32
	Doxycycline	16	R	32
	Tigecycline	0.25	S	4
Folate-pathway inhibitors	Trimethoprim/Sulfamethoxazole	>256	R	8
Aminoglycosides	Gentamicin	>256	R	>256
	Netilmicin	>256	R	>256
Phosphonic acids	Fosfomycin	>1024	R	64
Polymyxins	Colistin	0.125	S	128

**Fig 1:**
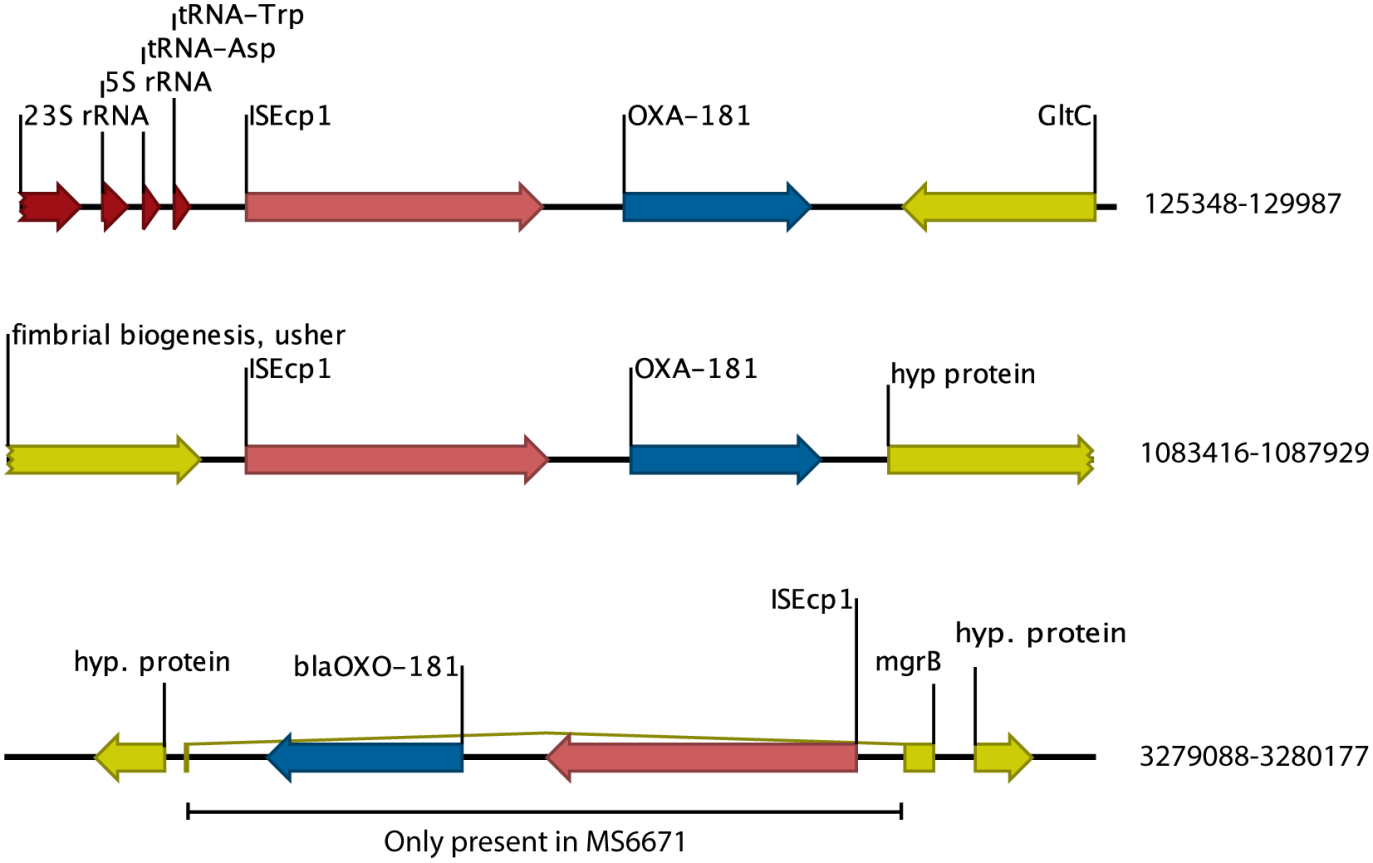
Comparison of blaOXA-181 insertions between DA48896 and MS6671. The OXA-181 reading frame is depicted in blue, the associated ISEcp1 is depicted in red, surrounding proteins coding genes are depicted in yellow and tRNA and rRNA genes in dark red. Positions indicated to the right are from the DA48896 genome.

**Fig 2:**
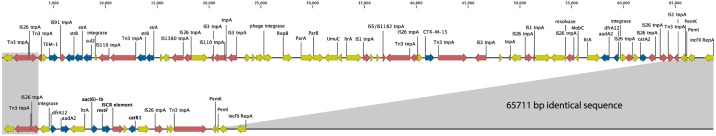
Comparison of resistance cassettes between pDA48896_1 and pMS6671_E. Resistance genes are depicted in blue, mobile genetic elements are depicted in red, surrounding proteins coding genes are depicted in yellow. The shaded areas depict identical sequences flanking the variable region. The plasmids contain a backbone of 69 kbp of highly similar sequence only shown for pMS6671 and not drawn to scale.

Whole genome sequencing was performed with both Pacific Biosciences and Illumina MiSeq techniques to generate a complete sequence of the genome and associated plasmids. The DA48896 strain was assigned to sequence type 147 and capsular serotype K64 and the chromosomal sequence had a very high sequence similarity to the previously sequenced *K*. *pneumoniae* isolate MS6671 (accession no. LN824133). MS6671 was isolated from a patient in the United Arab Emirates and resistant to all tested antibiotics including tigecycline and colistin, to which DA48896 was sensitive [22]. The chromosomes of the two strains were very closely related with only 46 individual nucleotide differences (S1 Table), 16 of which were clustered frame shifts restoring putatively frame-shifted reading frames compared to MS6671. These 16 differences occurred within a stretch of 2500 bp indicating either a recombinational event or previous sequence problems in this region. Furthermore, the chromosomes differed by 14 structural variations (insertions/deletions) the majority found in mobile genetic elements and intergenic regions (S2 Table). This high degree of sequence identity indicates a very recent common origin of the two isolates. The *bla*_OXA-181_ was found downstream of ISE*cp1* as reported previously. DA48896 has two copies of *bla*_OXA-181_ present on the chromosome at positions 127900 and 1086004, compared to 3 copies in MS6671 (Fig 1). Interestingly, the IS insertion disrupting the *mgrB* gene in MS6671 was missing in DA48896 explaining the difference in colistin susceptibility between the two strains. One copy of *bla*_CTX-M-15_ coupled to ISE*cp1* was present on the chromosome in the identical place as in MS6671 and a second copy of *bla*_CTX-M-15_ was carried on p48896_1, which also carried *bla*_TEM-1B_, *sul1*, *strA* and *strB* in a >70 kbp insertion compared to the otherwise very similar MS6671 plasmid E (LN824138) (Fig 2 and Table 2). This region was recently shown to vary extensively among other closely related OXA-181 producing ST147 isolates (see Discussion below) [23, 24]. DA48896 carried variants of 3 of the 5 plasmids of *K*. *pneumoniae* MS6671 (missing MS6671 plasmids A (LN824134) and C (LN824136)) but in addition three plasmids not found in MS6671 (Table 2). None of these extra plasmids contained any resistance genes. However, some plasmids contained larger replacements or rearrangements that affected the presence or locations of resistance genes (S3 Table).

**Table 2:** Plasmids found in DA48896.

Name	Size (bp)	Resistance genes	Plasmid replicons	Best match in MS6671	Best match in Genbank
p48896_1 (CP024430)	131,243	*aadA2*, *strAB*, *bla*_CTX-M-15_, *bla*_TEM-1_, *sul2*, *dfrA12*, *catA2*	IncFII, IncR	LN824138 (62% coverage/99% identity)	KT725788 (63% coverage/99% identity)
p48896_2 (CP024431)	114,815	*rmtf*, *aacA4*, *aac(6’)Ib-cr*	IncFII	LN824135 (87% coverage/99% identity)	CP021758 (100% coverage/99% identity)
p48896_3 (CP024432)	45,291	ND	ND	ND	CP011864 (91% coverage/98% identity)
p48896_4 (CP024433)	55,118	ND	ND	LN824139 (88% coverage/99% identity)	CP017988 (94% coverage/99% identity)
p48896_5 (CP024434)	4,644	ND	ND	LN824137 (100% coverage/100% identity)	LN824137 (100% coverage/100% identity)
p48896_6 (CP024435)	4,167	ND	ND	ND	EU932690 (100% coverage/100% identity)
p48896_7 (CP024436)	2,054	ND	*ColpVC*	ND	KU302803 (99% coverage/95% identity)

ND: Not detected.

Like MS6671, DA48896 was multi-resistant and carried an extensive number of resistance markers: *bla*_OXA-181_, *bla*_CTX-M-15_, *bla*_TEM-1_, *bla*_SHV-11_, *rmtf*, *aadA2*, *strAB*, *aacA4*, *oqxAB*, *aac(6’)Ib-cr*, *fosA*, *ARR-2*, *catA2*, *sul2* and *dfrA12*. DA48896 and MS6671 also differed by four SNPs in chromosomal genes previously known to affect resistance through efflux pumps, AcrR R18L, MarA V26D, S50G and RcsC L60M. BLASTp analysis showed that the mutation in AcrR (repressor of the AcrAB-TolC efflux pump) is unique to MS6671 as is the V26D mutation in MarA (multiple antibiotic resistance protein) while the MarA S50G and the RcsC (two-component sensor histidine kinase) L60M mutations were unique to DA48896. Examination of porin genes showed that both isolates carry an IS insertion associated with *bla*_CTX-M-15_ in the *ompk35* porin gene and an insertion of two additional amino acids in Ompk36. Also, a single non-synonymous mutation (Arg3Ser) was found in RamR, a transcriptional repressor of *ramA*. Inactivation of RamR has previously been shown to downregulate porin-expression and increase efflux resulting in antibiotic resistance.

## Discussion

DA48896 is a MDR *K*. *pneumoniae* belonging to the internationally spread clone ST147, and is very closely related to the pan-drug resistant *K*. *pneumoniae* MS6671 isolated in the UAE in 2014 [22]. Although they differed in several respects with regard to plasmid content, both isolates had almost the same antibiotic susceptibility profile except that DA48896 was sensitive to tigecycline and colistin. Colistin susceptibility could be explained by the absence of the IS insertion disrupting the *mgrB* gene in MS6671 [22]. It is intriguing that addition of more gene copies of the OXA-181 gene by replicative transposition, that likely is an adaptation to increased resistance to carbapenems, also has led to resistance to colistin, the last resort antibiotic for carbapenemases-producing multi-resistant *K*. *pneumoniae*. Furthermore, inactivation of RamR, a negative regulator of the *ramA* gene, leads to increased expression of *acrAB* which has been linked to tigecycline resistance [29]. MS6671 contain a non-synonymous mutation (Arg3Ser) in *ramR*, which was previously suggested to have caused tigecycline resistance in MS6671 [22]. However, DA48896 has the same non-synonymous mutation (Arg3Ser) in RamR but is susceptible to tigecycline. Instead, MS6671 contains a unique R187L mutation in AcrR, the repressor of *acrAB*, which could potentially give increased efflux through this pathway if it impedes the AcrR function. The otherwise very close relatedness between isolates DA48896 and MS6671 with very few SNPs suggests that strong selection for increased resistance to both these last resort antibiotics has occurred for MS6671 and indicates the direction of further resistance evolution in this internationally recognized pathogenic lineage. The genetic makeup and resistance profiles of the isolates indicate that the isolate found from Pakistan is an intermediate stage towards pan-resistance evolution of this particular clone. Mutations in *ompK36* present in both the isolates lead to generation of a previously described variant *ompk36v* with reduced influx of ertapenem [30]. *OmpK35* was also inactivated by IS insertion. The combination of multiple copies of *bla*_OXA-181_ and reduced permeability of both general porins may explain the high MIC of Ertapenem in these isolates.

Capsular serotype K64 has been reported as a major serotype associated with carbapenem resistance within the internationally widespread clone ST147 that is linked with a multitude of different virulence factors [31]. Presence of the Yersinia high-pathogenicity island and ABC transporter *kfu* in MS6671 and DA48896 makes these isolates likely to be hypervirulent [32]. The ST147 *K*. *pneumoniae* has previously been linked to the spread of ESBLs (especially CTX-M-15), VIM, OXA-48, KPC and recently also to NDM-1 in different clinical settings. The emergence of OXA-181 among this globally spread high-risk clone is worrying, leaving few therapeutic options available and contributing to a great impact in infection control measures [33, 34]. We screened 252 *K*. *pneumoniae* isolates from the same hospital over a period of 6 months and no further isolates with same *bla*_OXA-181_ genotype were recovered. In case of MS6671, no further isolates with the same genotype were found in the index patient’s hospital for 6 months [22]. Recently, closely related OXA-181 producing ST147 pan-drug resistant isolates have been described from 3 patients at a hospital in Abu Dhabi and a patient from USA [23, 24]. Complete whole genome sequences were not generated from these isolates but WGS plasmid data showed that they carried an IncFII plasmid with >66 kbp of identical backbone sequence as pMSS6671_E and pDA48896_1. However, the resistance regions of these plasmids also included *bla*_NDM-5_ and further varied extensively between the isolates illustrating the highly dynamic nature of resistance plasmids in this lineage. Finding such closely related ST147 isolates from multiple different geographical locations is of particular concern. OXA-181 is believed to have originated from the Indian sub-continent and bacteria harbouring this gene have been reported frequently from this region [10, 13, 15].

## Conclusions

In conclusion, to the best of our knowledge, we report the first OXA-181 producing ST147 *K*. *pneumoniae* from Pakistan. Its close relatedness to MS6671 from UAE gives an insight into the role of ST147 in OXA-181 dissemination, the rapid evolution of resistance towards the two last resort antibiotics, colistin and tigecycline, and global transfer of this international risk clone.

## Supporting information

S1 Table Single nucleotide polymorphisms between DA48896 and MS6671. (XLSX)

S2 Table Chromosomal structural variations between DA48896 and MS6671. (XLSX)

S3 Table Structural variations between plasmids. (XLSX)
